# Supplementary material for: The hepatic AMPK-TET1-SIRT1 axis regulates glucose homeostasis
Source: eLife. 2021 Nov 5;10:e70672. doi: 10.7554/eLife.70672 (PMC8592569; doi:10.7554/eLife.70672)
Supplement: Figure 5—source data 1. [file elife-70672-fig5-data1.pdf]

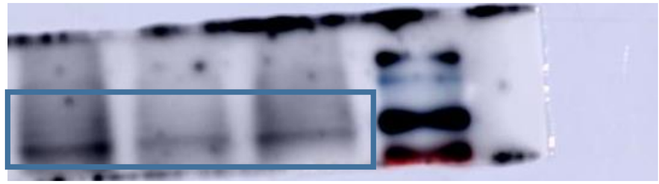
Original Data is used for Figure 5J
Line 1

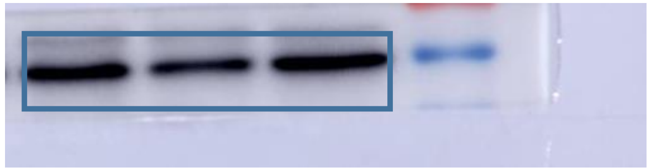
Original Data is used for Figure 5J
Line 2

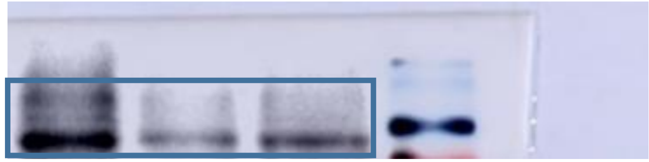
Original Data is used for Figure 5J
Line 3

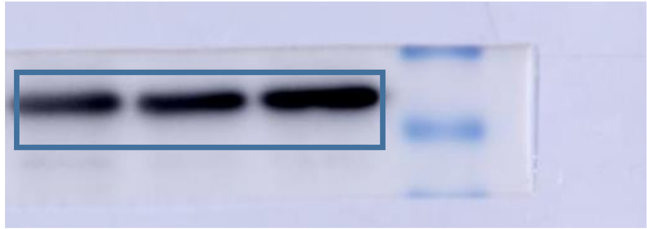
Original Data is used for Figure 5J
Line 4
