# Supplementary material for: The hepatic AMPK-TET1-SIRT1 axis regulates glucose homeostasis
Source: eLife. 2021 Nov 5;10:e70672. doi: 10.7554/eLife.70672 (PMC8592569; doi:10.7554/eLife.70672)
Supplement: Figure 5—figure supplement 1—source data 1. [file elife-70672-fig5-figsupp1-data1.pdf]

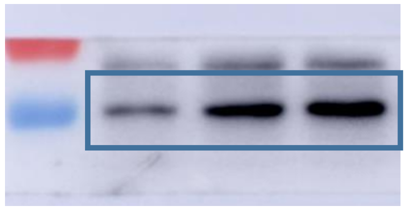

Original Data is used for Figure 5 Supplement 1C Line 1

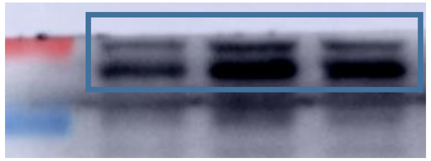

Original Data is used for Figure 5 Supplement 1C

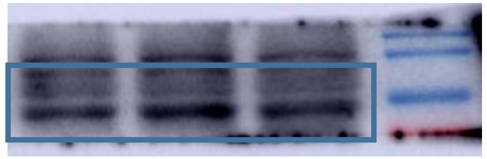

Original Data is used for Figure 5 Supplement 1C Line 3

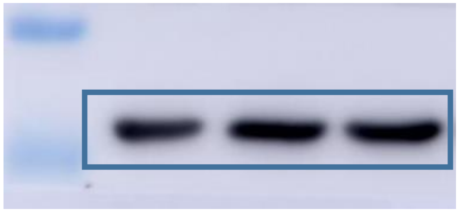

Original Data is used for Figure 5 Supplement 1C Line 4
