# Supplementary material for: The hepatic AMPK-TET1-SIRT1 axis regulates glucose homeostasis
Source: eLife. 2021 Nov 5;10:e70672. doi: 10.7554/eLife.70672 (PMC8592569; doi:10.7554/eLife.70672)
Supplement: Supplementary file 1. [file elife-70672-supp1.docx]

**Supplementary file 1. List of primers**

| **Cloning primers** |
| --- |
| \| TET1 1-654aa. S \| CCGCTCGAGATGTCTCGATCCCGCCATGCAAGG \| \| --- \| --- \| \| TET1 1-654aa. A \| CCCTCTAGATCAAACTTTGGGCTTCTTTTCCCT \| \| TET1 620-1590aa. S \| CCGCTCGAGATGAAGAAAAGAAAATGTGAGGAG \| \| TET1 620-1590aa. A \| CCCTCTAGATCAAAACTTACAGCCATTAAAGTA \| \| TET1 1418-2138aa. S \| GCTGAATTCATGGAACTGCCCACCTGCAGCTGTC \| \| TET1 1418-2138aa. A \| CCCTCTAGATCAGACCCAATGGTTATAGGGCCC \| |
| **Real time PCR primers** |
| \| *G6PC*. S \| GCAGGTGTATACTACGTGATGGT \| \| --- \| --- \| \| *G6PC*. A \| GACATTCAAGCACCGAAATCTG \| \| *PPARGC1A*. S \| TGACTGGCGTCATTCAGGAG \| \| *PPARGC1A*. A \| CCAGAGCAGCACACTCGAT \| \| *SLC2A4*. S \| GGGAAGGAAAAGGGCTATGCTG \| \| *SLC2A4*. A \| CAATGAGGAACCGTCCAAGAATG \| \| *TET1*. S \| GGAATGGAAGCCAAGATCAA \| \| *TET1*. A \| ACTCCCTAAGGTTGGCAGTG \| \| *PCK1*.S \| ATCCCCAAAACAGGCCTCAG \| \| *PCK1*. A \| ACGTACATGGTGCGACCTTT \| \| *ACTB*. S \| TACTGCCCTGGCTCCTAGCA \| \| *ACTB*. A \| GCCAGGATAGAGCCACCAATC \| \| *Sirt1*. S \| CAGACCCTCAAGCCATGTTT \| \| *Sirt1*. A \| CTTTGGATTCCTGCAACCTG \| \| *Sirt6*. S \| ATCTTCGACCCACCAGAGG \| \| *Sirt6*. A \| GCATTCTCGAAGGTGGTGTC \| \| *Tet1*. S \| GACCCTCATAAGCAGAGAGGAAA \| \| *Tet1*. A \| TGACACCAGAGAAAGGACGC \| \| *G6pc*. S \| AAGAGCGCAACAGTTCCCTT \| \| *G6pc*. A \| CTCTGGCCTCACAATGGGTT \| \| *Slc2a4*. S \| GGGAAGGAAAAGGGCTATGCTG \| \| *Slc2a4*. A \| CAATGAGGAACCGTCCAAGAATG \| \| *Ppargc1a*. S \| TCTCAGTAAGGGGCTGGTTG \| \| *Ppargc1a*. A \| AGCAGCACACTCTATGTCACTC \| \| *Actb*. S \| CTACCTCATGAAGATCCTGACC \| \| *Actb*. A \| GAACCGCTCGTTGCCAATAGTG \| \| *ALDH1A3*. S \| GGGCCTCAGATCGACCAAAA \| \| *ALDH1A3*. A \| GCTGCACTGGTCCGAAAATC \| \| *ALDOA*. S \| AGCTGAATAGGCTGCGTTCT \| \| *ALDOA*. A \| GACAGGCGGGTCATGTTGAA \| |
|  |
|  |

**hMeDIP PCR primers**

*G6PC*.S ATGGCCGATCAGGCTGTTTT

*G6PC*.A CCAGCCCTGATCTTTGGACT

*PPARGC1A*.S GATTGGCAATGGGAGCAACC

*PPARGC1A*.A AGACACATCGTGCTGTCAGG

*SLC2A4*.S AACTTGGCAATTGGGGCAAC

*SLC2A4*.A GCTGCCACTTCAGTCCAGAT
